# Severe Dermatophytosis and Acquired or Innate Immunodeficiency: A Review

**DOI:** 10.3390/jof2010004

**Published:** 2015-12-31

**Authors:** Claire Rouzaud, Roderick Hay, Olivier Chosidow, Nicolas Dupin, Anne Puel, Olivier Lortholary, Fanny Lanternier

**Affiliations:** 1 Centre d’Infectiologie Necker-Pasteur, Hôpital Necker Enfants Malades et Institut Imagine, APHP, Université Paris Descartes, Sorbonne Paris Cité, 75015 Paris, France; olivier.lortholary@aphp.fr; 2 Dermatology Department, King’s College Hospital NHS Trust, London SE5 9RS, UK; roderick.hay@ifd.org; 3 Service de Dermatologie, Hôpital Henri Mondor, APHP, Université Paris-Est Créteil, 94010 Créteil, France; olivier.chosidow@aphp.fr; 4 Service de Dermatologie, Hôpital Cochin, APHP, Université Paris Descartes, Sorbonne Paris Cité, 75014 Paris, France; nicolas.dupin@aphp.fr; 5 Laboratoire de Génétique Humaine des Maladies Infectieuses, INSERM U1163, Hôpital Necker Enfants Malades et Institut Imagine, Université Paris Descartes, Sorbonne Paris Cité, 75015 Paris, France; anne.puel@inserm.fr; 6 Institut Pasteur, Centre National de Référence Mycoses Invasives, 75015 Paris, France; 7 Institut Pasteur, Unité de Mycologie Moléculaire, CNRS URA3012, 75015 Paris, France

**Keywords:** dermatophytosis, *Trichophyton rubrum*, immunodepression, organ transplant, HIV, CARD9 deficiency

## Abstract

Dermatophytes are keratinophilic fungi responsible for benign and common forms of infection worldwide. However, they can lead to rare and severe diseases in immunocompromised patients. Severe forms include extensive and/or invasive dermatophytosis, *i.e.*, deep dermatophytosis and Majocchi’s granuloma. They are reported in immunocompromised hosts with primary (autosomal recessive CARD9 deficiency) or acquired (solid organ transplantation, autoimmune diseases requiring immunosuppressive treatments, HIV infection) immunodeficiencies. The clinical manifestations of the infection are not specific. Lymph node and organ involvement may also occur. Diagnosis requires both mycological and histological findings. There is no consensus on treatment. Systemic antifungal agents such as terbinafine and azoles (itraconazole or posaconazole) are effective. However, long-term outcome and treatment management depend on the site and extent of the infection and the nature of the underlying immunodeficiency.

## 1. Introduction

Fungal infections of the skin and nails are frequent. They affect 20% to 25% of the world’s population and represent the fourth most prevalent of human diseases [1,2,3]. Dermatophytes account for the largest group of pathogens responsible for skin mycoses. Dermatophytes are cosmopolitan, keratinophilic filamentous fungi belonging to the genera *Trichophyton*, *Microsporum* and *Epidermophyton*. They usually cause benign and common infections limited to the stratum corneum or keratinized adnexal structures, such as tinea capitis, tinea corporis, tinea pedis, tinea cruris, tinea unguium [4]. However, dermatophytes can also be responsible for extensive or invasive forms in immunocompromised hosts. These severe forms of dermatophytosis are still frequently not recognized by clinicians. Their health impact may be due to their extensive nature or to deep penetration including dermal invasion (deep dermatophytosis and Majocchi’s granuloma).

The aim of this review is to describe the physiopathology, epidemiology and the different forms of severe dermatophytosis in immunocompromised hosts including their clinical presentation, the diagnostic strategy and treatment.

## 2. Physiopathology

Geophilic dermatophyte species survive in the environment where there is contact with keratinized material, anthropophilic dermatophytes only infect humans and zoophilic dermatophytes infect animals and, occasionally, humans. Arthrospores present in the environment or shed skin scales are responsible for inoculation. This morphological form of the dermatophyte can survive several months outside the host [5]. After inoculation, arthrospores adhere to the keratinocytes, a process that occurs within 2 h [6]. Proteases such as the subtilisins secreted by dermatophytes play a key role in this and the penetration phase. Dermatophytes also produce sulphites and reducing agents to allow proteases to degrade keratin, which serves as a nutrient. The secreted proteases have been also identified as virulence factors. Once they have adhered to human keratinocytes, dermatophytes penetrate into the stratum corneum [7]. This process may be facilitated by an alteration in the structure or environment of the stratum corneum (humidity, trauma). Stratum corneum and hair follicular ostium environment provide the dermatophyte’s nutritional and pH requirements. A number of locally produced substances, such as β defensins, unsaturated transferrin contained in sweat and sebum, and unsaturated long chain fatty acids produced by the sebaceous glands, inhibit the dermatophyte’s growth in the epidermis.

Animal models and clinical data provide evidence that, under certain circumstances, dermatophytes can invade the dermis, survive and spread to lymph nodes and internal organs. Indeed, subcutaneous inoculation of *Trichophyton mentagrophytes* in mice, led to dissemination to lymph nodes, liver and spleen [8]. The possible role of lymphatic spread has been discussed in relation to proximal white subungual onychomycosis and in cases of deep dermatophytosis in humans [9,10]. An infection appearing in the region of the nail lunula may indicate endogenous reactivation or autoreinfection from a deeper site rather than a new external infection. In CARD9 (caspase recruitment domain-containing protein 9) deficient patients, dermatophytes are able to disseminate to lymph nodes [11]. 

The skin represents the first barrier against dermatophytes where they come into contact with Langerhans cells [12]. Both innate and adaptive immunities play a role in dermatophyte antifungal defense. The C-type lectin receptors, such as DECTIN-1, DECTIN-2 or MINCLE are involved in the recognition of dermatophytes in mice models. DECTIN-2 preferentially recognizes hyphae and DECTIN-1 conidia of *T. rubrum* [13,14]. This recognition induces an intracellular signaling cascade with the CARD9 adaptor protein playing a central role [11] and induces the production of pro-inflammatory cytokines responsible for the recruitment of immune cells to the site of infection. The role of T lymphocytes in anti-dermatophytes immunity has been studied in animal models: transfer of mouse lymphocytes infected by dermatophytes in sub-lethally irradiated mice protected them from infection [15]. It was also found that *T. rubrum* conidia are ingested by peritoneal mouse macrophages within four hours which in turn produce TNF-α and IL-10. After 8 h of infection, conidia produce hyphae inside macrophages [16]. The dermatophytes can also develop virulence factors that enable them to evade or suppress host-defense. *T. rubrum* is the best adapted to human skin and generally causes chronic and non-inflammatory lesions [8,10].

## 3. Mycology

Dermatophytes are a group of filamentous fungi mainly characterized by their ability to grow in the presence of keratin substrates within their immediate environment. There are three genera of dermatophytes: *Epidermophyton*, *Microsporum* and *Trichophyton*. All dermatophytes are ascomycetous, members of the class *Euascomycetes*. 

Many dermatophyte species have been reported to be responsible for infections. *T. rubrum* is the most common dermatophyte species worldwide [1]. Dominance of a particular dermatophyte species depends on the clinical localization and geographical area. *T. violaceum*, *T. tonsurans*, *T. soudanense*, *Microsporum canis*, *M. audouinii* are the dominant species found in scalp infections [2]. The anthropophilic dermatophytes *M.* *audouinii*, *T. violaceum*, *T. tonsurans* and *T. soudanense* are the most prevalent pathogens in Africa [2]. Several dermatophyte species such as *T. concentricum* are geographically constrained but sporadic infections outside their principal regional sources are possible, probably related to international travel [5].

*T. rubrum* is the species most often involved in severe dermatophytosis [17,18] although *M. canis*, *T. tonsurans*, *T. mentagrophytes*, *T. violaceum* and *Epidermophyton flocosum* have also been reported in some cases [11,17,19]. Species distribution in severe dermatophytosis has the same epidemiology and worldwide distribution as benign dermatophytosis. *T. rubrum* is the most common dermatophyte species worldwide in severe dermatophytosis cases but *T. violaceum* is more frequent in Africa. These are the species reported in the patients from North Africa with CARD9 deficiency and deep dermatophytosis. Therefore, no one species appears to be specifically associated with severe forms of the disease.

## 4. Different Forms of Dermatophytosis

Superficial infections caused by dermatophytes limited to the stratum corneum are the most common clinical presentation. In contrast, severe dermatophytosis is a rare and poorly described entity, including different forms *i.e.*, invasive forms of infection (deep dermatophytosis and Majocchi’s granuloma) and extensive dermatophytosis.

Different forms of severe dermatophytosis:

Invasive dermatophytosis

- Localised to single or multiple perifollicular (hair follicle) sites e.g., Majocchi granuloma, nodular perifolliculitis.
- Deep dermatophytosis, not confined to the perifollicular area e.g., in the presence of immunosuppression or CARD9 deficiency, with or without dissemination to extra-cutaneous sites.

Extensive dermatophytosis is an infection confined to the stratum corneum but with unusually extensive or numerous lesions.

**Figure 1:**
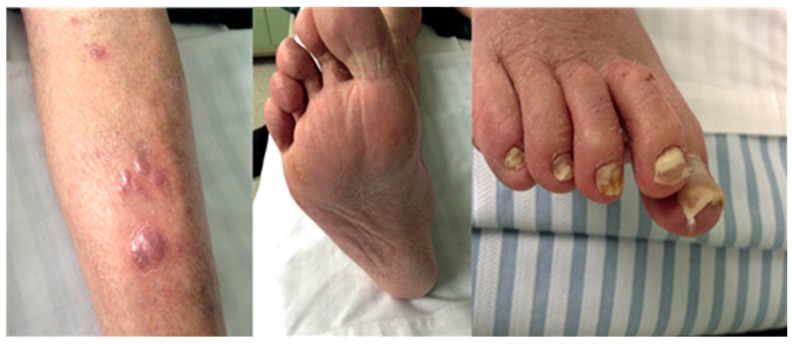
Purple nodules on the leg, interdigital and scaly lesions of the foot, proximal and total diffuse onychomycosis in a solid organ transplant patient diagnosed with deep dermatophytosis caused by *T. rubrum*.

Invasive dermatophytosis is defined by the presence of dermal invasion in two clinical forms, Majocchi’s granuloma or deep dermatophytosis. Majocchi’s granuloma, first described in 1883 [20], is a limited perifollicular granuloma. Its histological characteristic is perifollicular granulomatous inflammation with dermal dermatophyte abscesses. Clinically, lesions appear as nodules or papules on the lower limb or head and the granuloma follows inflammatory rupture of the hair follicle with transfer of the dermatophyte hyphae into the dermis. It may also be called nodular folliculitis or perifolliculitis [21]. In contrast, deep dermatophytosis is characterized by the extension of infection beyond the perifollicular area [11]. The lesions appear as ill-defined infiltrated plaques, nodules and papules sometimes associated with itching, pain, and discharge (Figure 1). The number and location of lesions are variable [17,22]. In this form, the dermatophyte infection can sometimes spread to lymph nodes and other organs either by contiguity (e.g., bone) or through vascular or lymphatic spread (e.g., central nervous system) [11,23,24]. One clue to the diagnosis of these atypical forms is the presence of associated typical superficial dermatophytosis lesions such as onychomycosis that may provide a nidus of infection for subsequent dissemination [19,25].

Extensive dermatophytosis is characterized either by the unusual extent of skin surface area affected by the infection or by the unusual number of affected sites [26,27,28,29]. However, the infection remains confined to the epidermis or associated keratinized structures such as nails.

## 5. Diagnosis of Severe Dermatophytosis

**Figure 2:**
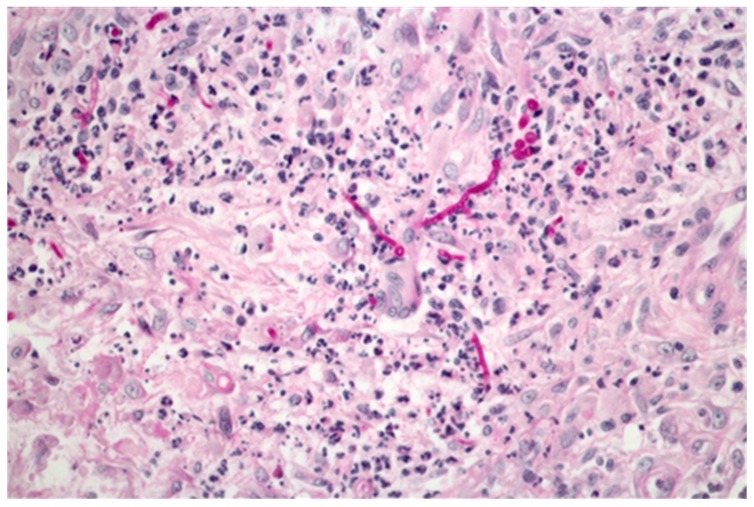
Skin-biopsy specimen from an organ transplant patient with deep dermatophytosis (PAS, 400× ) showing hyphae in the dermis.

Cutaneous manifestations of the infection are not sufficiently specific and the presentation may be polymorphic, therefore clinical diagnosis can be difficult [29,30]. Biopsy for histopathology and culture is required for diagnosis. The diagnosis of invasive dermatophytosis is confirmed by the presence of hyphae compatible with dermatophytes *i.e.*, short thick, and sometimes irregular, septate hyphae (Figure 2) in the dermis and a positive culture for a dermatophyte.

A series of 17 cases, diagnosed as Majocchi’s granuloma, showed variable acanthosis in most biopsies suggesting initial involvement of the epidermis [31]. The dermis was infiltrated by lymphohistiocytic cells and neutrophils. Capillary proliferation, vascular hyperplasia and extravasation of red blood cells were present in all samples with, in some samples, fibrinoid change in the blood vessels. Rupture or disintegration of the hair follicle was present in all biopsies. PAS (Periodic Acid Schiff) and GMS (Grocott’s Methenamine Silver) staining showed fungal elements in the keratin layer in some cases and in the dermis in all cases. Biopsies from CARD9 deficient patients with deep dermatophytosis showed deep granulomas in 10 of the 17 patients, with necrosis in 6 of them [11].

Immunocompromised patients can also develop non-dermatophyte mold infections of the skin associated with presence of hyphae; these include aspergillosis, mucormycosis, fusariosis or phaeohyphomycosis. Therefore, culture is necessary to confirm diagnosis of deep dermatophytosis and to identify the pathogen. In the absence of positive culture, immunohistochemistry with specific antibodies against species such as *T. rubrum* may also be performed or specific dermatophyte molecular diagnostic tools applied to tissue samples may help to confirm the diagnosis [25].

## 6. Underlying Conditions Responsible for Severe Dermatophytsosis

The various forms of severe dermatophytosis, as defined above, may occur in different groups of patients, most of whom have identifiable innate or acquired immunodeficiency. The literature review was conducted via the Pubmed database (Table 1).

**Table 1:** Underlying conditions associated with severe dermatophytosis, a review of the literature.

Underlying Conditions Associated with Severe Dermatophytosis	Number of Cases	Clinical Involvement	References
Solid organ transplant	28	Skin	[22,26,27,31,32,33,34,35,36,37,38,39,40,41,42,43,44,45,46,47,48,49,50,51,52,53]
HIV infection	9	Skin	[38,54,55,56,57,58,59,60]
Systemic corticosteroid treatment	7	Skin	[21,31,61,62,63,64,65]
Others immunosuppressive treatments ± steroid	6	Skin/nodes	[19,25,66,67,68,69]
Hematological malignancy	7	Skin	[70,71,72,73,74]
Liver disease	3	Skin	[17,75,76]
Topical steroid only	1	Skin/nodes	[77]
Cushing disease, congenital adrenal hyperplasia	2	Skin	[78,79]
Atopy	1	Skin	[80]
Diabetes mellitus	1	Skin	[64]
CARD9 deficiency	19	Skin/nodes/organs	[11,23,81,82,83,84,85,86,87]

### 6.1. Solid Organ Transplant

Since 1987, 28 cases of severe, most often invasive dermatophytosis in solid organ transplant recipients have been published [22,26,27,31,32,33,34,35,36,37,38,39,40,41,42,43,44,45,46,47,48,49,50,51,52,53]. Most of these patients were males (20/28), kidney or heart transplant recipients. The median age was 49 years [14,15,16,17,18,19,20,21,22,23,24,25,26,27,28,29,30,31,32,33,34,35,36,37,38,39,40,41,42,43,44,45,46,47,48,49,50,51,52,53,54,55,56,57,58,59,60,61,62,63,64,65,66,67,68]. The time reported between the diagnosis of severe dermatophytosis and transplantation varied (1 to 192 months). The clinical presentation was not specific but the authors described nodules on the lower limbs, sometimes together with dermatophyte infections of nails, groins and/or other skin sites. *T. rubrum* was the species most frequently involved. Prolonged antifungal treatment was effective and notably there were no deaths reported. Terbinafine and itraconazole were the most commonly used antifungal agents.

### 6.2. HIV Infection

Nine cases of severe dermatophytosis in HIV infected patients have been reported in the literature between 1999 and 2004 [38,54,55,56,57,58,59,60]. One patient had received corticosteroids, and four patients were known to be intravenous drug users. The CD4 counts were between 16 and 335/mm^3^. Dermatophytosis was extensive in three patients. Dermatophyte cultures were positive, however no biopsy data are available for these three patients. Six patients had confirmed deep infections. The lesions, consistent with cutaneous dermatophyte infection, were present at the time of diagnosis of HIV infection in one patient. The lesions were always multiple and described as erythematous, scaly, circular plaques and/or erythematous papules or nodules. The trunk, arms, legs and even the face were affected. Onychomycosis was noted in two patients. *T. rubrum* (2), *M. canis* (2), *M. gypseum* (2), *T. mentagrophytes* (2), *T. tonsurans* (1), *M. gallinae* (1), were the dermatophyte species involved. Four patients received topical antifungal treatment including two of with associated systemic antifungals. The systemic antifungals prescribed were: ketoconazole, fluconazole, terbinafine or itraconazole. The treatment duration was variable and three patients died due to other complications.

### 6.3. Other Secondary Immunodeficiencies

Among the cases of severe dermatophytosis with microbiological confirmation, 28 have been reported in the English language literature since 1979 [17,19,21,25,31,61,62,63,64,65,66,67,68,69,70,71,72,73,74,75,76,77,78,79,80]. The patients were mainly men [19] aged from 15 to 75 years. In two patients, dermatophytosis was extensive [19] and invasive in the remaining cases. In three patients (atopic dermatitis receiving topical steroids (1) or immunosuppressive treatment for myasthenia (1) or autoimmune hepatitis (1)), there was a proven lymph node infection [77] or lymph node or deep organ involvement was suspected [67,68]. In this series of cases predisposing conditions were: systemic corticosteroid treatment (*n* = 13), topical steroid (*n* = 2), myelodysplastic syndrome, leukemia or lymphoma (*n* = 7), atopic dermatitis and eczema (*n* = 3), diabetes mellitus (*n* = 3), alcoholic cirrhosis and hereditary haemochromatosis with liver failure (*n* = 1), hepatitis B and C related cirrhosis and haemodialysis for renal failure (*n* = 1), alcoholic liver disease (*n* = 1), Cushing disease (*n* = 1), congenital adrenal hyperplasia (*n* = 1). Some patients had been receiving immunosuppressive drugs: azathioprine (*n* = 3), cyclosporine (*n* = 2), cyclophosphamide, methotrexate, infliximab, tacrolimus and topical tacrolimus (*n* = 1) mainly for autoimmune disease (myasthenia (*n* = 2), autoimmune hepatitis (*n* = 2), lupus (*n* = 2), rheumatoid arthritis and Behçet’s disease (*n* = 1)). Most patients had more than one risk factor for infection. In this group of patients, *T. rubrum* was the predominant species. *M. canis*, *T. mentagrophytes*, *T. violaceum*, *T. verrucosum* and *E. flocosum* were sometimes involved. Severe dermatophytosis presented with nodules, papules, abscesses and erythematous plaques. Isolated lesions were rare. The arms, legs, face and trunk were also affected. In nine cases, superficial dermatophyte lesions were also noted. Two patients received topical treatment. One patient, with alcoholic liver disease, was initially treated with topical terbinafine but subsequently received oral terbinafine 250 mg/day because of a lack of efficacy of topical therapy. The second patient was successfully managed with local injections of miconazole (the intravenous formulation). Surgery, including surgical excision at the time of diagnosis, local excision of a nodule or, in one case, drainage followed by instillation of amphotericin B into the large cysts prior to subsequent surgical removal, was performed in four patients. At the time of dermatophytosis diagnosis, the initial systemic antifungal treatment was terbinafine (*n* = 6), itraconazole (*n* = 6), ketoconazole (*n* = 5), fluconazole (*n* = 3) and griseofulvin (*n* = 3). Two patients relapsed [64,77] and several patients died due to underlying comorbidities.

### 6.4. Primary Immunodeficiency

CARD9 deficiency predisposes to severe dermatophytosis, mainly deep dermatophytosis [88]. A genetic origin was long suspected to predispose to this disease, also called dermatophytic disease [89]. The first cases of dematophytic disease were reported among Japanese and North African individuals. However, recently, 19 patients with deep dermatophytosis without any known risk factors were reported to have autosomal recessive CARD9 deficiency [11,23,81,82,83,84,85,86,87]. Among these, there was a predominance of male patients (14 out 19 cases). Most patients were from known consanguineous unions. The patients originated from Tunisia, Algeria, Morocco, Egypt and Brazil. Clinically, the lesions started in childhood with recurrent and extensive superficial lesions. In early adulthood, these patients developed extensive erythematous scaly lesions, subcutaneous nodules, or infiltrated ulcerated lesions and fistulae. Lesions were recurrent. Almost all patients had more typical lesions of tinea corporis (ringworm) (16/19) and onychomycosis (15/19). The most frequently isolated dermatophyte species were *T. violaceum* and *T. rubrum*. Lymph node involvement was present in 10 patients and organ involvement by contiguity in 2 patients. Lesions relapsed after discontinuation of antifungal treatment. These patients required lifelong anti-dermatophyte maintenance therapy. CARD9 deficiency only predisposes to fungal infections. Other fungal diseases such as *Candida* colitis and central nervous system infection or phaeohyphomycosis have been reported in CARD9 deficient patients [88].

Extensive dermatophytosis with hyperkeratotic dermatophytosis confined to the stratum corneum may also occur in patients with chronic mucocutaneous candidiasis associated with *STAT1* gain-of-function mutations [90,91]. Some patients with extensive dermatophytosis have an underlying disorder of keratinisation such as the KID (Keratitis, Ichthyosis, Deafness) syndrome [92].

Some published cases were compatible with deep or extensive dermatophytosis but lacked mycological confirmation [31,93,94]. In other published cases, there was no identified predisposing condition [28,95,96,97,98,99,100,101,102,103,104,105,106,107,108,109,110]. However underlying immunodeficiency cannot be excluded in these patients without thorough immunological investigations.

## 7. Treatment of Severe Dermatophytosis

Currently, there is no consensus treatment for extensive or invasive dermatophytosis. Systemic antifungals active *in vitro* against dermatophytes are griseofulvin, terbinafine, ketoconazole, fluconazole, itraconazole, posaconazole, voriconazole, ravuconazole [111,112]. An *in vitro* study tested five antifungal agents against 129 dermatophyte strains belonging to 12 different species. Drugs showing antifungal activity, from more to least antifungal activity, were: terbinafine, posaconazole, ravuconazole, itraconazole and fluconazole [111]. The new azole isavuconazole could be useful against *Trichophyton* and *Epidermophyton* but clinical effectiveness has yet to be determined [113].

In recent reported cases of severe dermatophytosis, successes were obtained with triazoles such as posaconazole or itraconazole [53,81]. In a review, Marconi *et al.* [17] reported successful treatment with terbinafine and itraconazole. One other study also describes the use of terbinafine as this antifungal is associated with a good penetration into the stratum corneum [114]. Another investigation demonstrated adequate skin penetration of posaconazole in 30 healthy adult human subjects receiving posaconazole by oral suspension [115]. Cases of treatment failure (with topical or systemic treatment) and relapses have also been reported [11,22,35,81,116]. Treatment duration depends on the response and predisposing factors. For patients with CARD9 deficiency, a rebound effect upon discontinuation of antifungal therapy has been observed. Thus, secondary lifelong prophylaxis is suggested [11]. In practice, terbinafine (250 mg per day) is usually recommended as a first-line treatment, unless the cause is a *Microsporum* species against which an azole is more appropriate. This treatment requires regular monitoring of liver function. Posaconazole may be an alternative in cases that do not respond. The plasma concentration of this antifungal should be monitored, and posaconazole tablets are the preferred formulation for optimal plasma concentration. Itraconazole is another alternative. Management of azole interactions with immunosuppressive medications (e.g., cyclosporin) and side effects due to prolonged use (neuropathy) form a key part of the process of monitoring the use of therapy. Topical antifungal therapy may in addition be given in particular for superficial lesions, such as amorolfine for onychomycosis. Surgical treatment has been reported for highly localized lesions [17,117,118].

## 8. Conclusions

Dermatophytes are common fungal pathogens that mainly cause superficial infections of the skin, nails, and hair. In immunocompromised patients, they can cause atypical infections, with unusually extensive lesions or dermal invasion. The diagnosis of severe either invasive or extensive dermatophytosis without clearly identifiable risk factors should lead to screening for inherited immunodeficiencies, such as CARD9 deficiency. The prevalence of these severe forms may be underestimated. Their low frequency contrasts with high prevalence of dermatophytosis worldwide. In the future, a better knowledge of the frequency and description of this entity in different groups of immunosuppressed patients will help to improve our understanding of the pathogenesis of dermatophytosis and to develop new therapeutic options. Treatment of superficial dermatophytosis by prompt and adequate treatment when starting immunosuppressive treatment is an important step in preventing the development of the more severe forms of disease.
